# Progression-free survival at 2 years is a reliable surrogate marker for the 5-year survival rate in patients with locally advanced non-small cell lung cancer treated with chemoradiotherapy

**DOI:** 10.1186/1471-2407-14-18

**Published:** 2014-01-14

**Authors:** Hiroaki Akamatsu, Keita Mori, Tateaki Naito, Hisao Imai, Akira Ono, Takehito Shukuya, Tetsuhiko Taira, Hirotsugu Kenmotsu, Haruyasu Murakami, Masahiro Endo, Hideyuki Harada, Toshiaki Takahashi, Nobuyuki Yamamoto

**Affiliations:** 1 Division of Thoracic Oncology, Shizuoka Cancer Center, Shimonagakubo, 1007 Shimonagakubo, Nagaizumi-cho Sunto-gun, Shizuoka 411-8777, Japan; 2 Clinical Trial Management Department, Shizuoka Cancer Center, Shimonagakubo, Nagaizumi-cho Sunto-gun, Shizuoka, Japan; 3 Department of Respiratory Medicine, Juntendo University, Hongou Bunkyou-ku, Tokyo, Japan; 4 Division of Diagnostic Radiology, Shizuoka Cancer Center, Shimonagakubo, Nagaizumi-cho Sunto-gun, Shizuoka, Japan; 5 Division of Radiation Oncology, Shizuoka Cancer Center, Shimonagakubo, Nagaizumi-cho Sunto-gun, Shizuoka, Japan; 6 Third Department of Internal Medicine, Wakayama Medical University, Kimiidera, Wakayama, Japan

**Keywords:** Locally advanced non-small cell lung cancer, Chemoradiotherapy, Surrogate endpoint, Overall response rate, Progression-free survival

## Abstract

**Background:**

In locally advanced Non-Small-Cell Lung Cancer (LA-NSCLC) patients treated with chemoradiotherapy (CRT), optimal surrogate endpoint for cure has not been fully investigated.

**Methods:**

The clinical records of LA-NSCLC patients treated with concurrent CRT at Shizuoka Cancer Center between Sep. 2002 and Dec. 2009 were reviewed. The primary outcome of this study was to evaluate the surrogacy of overall response rate (ORR) and progression-free survival (PFS) rate at 3-month intervals (from 9 to 30 months after the initiation of treatment) for the 5-year survival rate. Landmark analyses were performed to assess the association of these outcomes with the 5-year survival rate.

**Results:**

One hundred and fifty-nine patients were eligible for this study. The median follow-up time for censored patients was 57 months. The ORR was 72%, median PFS was 12 months, and median survival time was 39 months.

Kaplan-Meier curve of progression-free survival and hazard ratio of landmark analysis at each time point suggest that most progression occurred within 2 years. With regard to 5-year survival rate, patients with complete response, or partial response had a rate of 45%. Five-year survival rates of patients who were progression free at each time point (3-months intervals from 9 to 30 months) were 53%, 69%, 75%, 82%, 84%, 89%, 90%, and 90%, respectively. The rate gradually increased in accordance with progression-free interval extended, and finally reached a plateau at 24 months.

**Conclusions:**

Progression-free survival at 2 years could be a reliable surrogate marker for the 5-year survival rate in LA-NSCLC patients treated with concurrent CRT.

## Background

Lung cancer is the most common type of cancer, both worldwide and in Japan [1]. Non-small cell lung cancer (NSCLC) accounts for 80-85% of lung cancer cases, and approximately 30% of patients have unresectable, locally advanced disease at diagnosis [2]. In the 1990’s, radiotherapy alone was recognized as the standard treatment, but its efficacy was insufficient [3]. Sause et al., reported that adding chemotherapy to radiotherapy brought further survival benefit [4]. A recent meta-analysis concluded that concurrent chemoradiotherapy (CRT) is state-of-the art treatment in this population [5,6].

The goal of CRT in locally advanced NSCLC (LA-NSCLC) is to cure. In the early period of treatment, tumor shrinkage is an indicator of efficacy. Although concurrent CRT provides a high rate of tumor response (60–70%), we should take into account that it does not always mean cure. Recent phase III trials of concurrent CRT reported that two-thirds of patients who experienced complete, or partial response eventually relapsed [7,8]. Another indicator of efficacy is progression-free survival (PFS). The Kaplan-Meier curves of PFS in LA-NSCLC showed the “infant mortality” type. This means that most progression occurred in the first 2 to 3 years. Therefore, we speculate that PFS rate at 2 years could be another candidate surrogate for cure.

Overall survival (OS) is the gold standard endpoint in phase III trials. However, it requires long-term follow-up, and a large number of patients. Overall response rate (ORR), median PFS, and PFS rate at specific time points were commonly adopted primary endpoints in phase II trials. However, their surrogacy for cure has not been fully investigated. The aim of this study is to search for the optimal surrogate marker of the 5-year survival rate in patients with LA-NSCLC treated with CRT.

## Methods

### Patient selection and treatment methods

We collected the clinical records of LA-NSCLC patients treated with concurrent CRT at Shizuoka Cancer Center between Sep. 2002 and Dec. 2009. The eligibility criteria of this study was as follows: (1) histologically or cytologically proven NSCLC; (2) chemoradiotherapy naïve; (3) age < 75 years; (4) Eastern Cooperative Oncology Group Performance Status (ECOG PS) of 0 to 2; and (5) treated with curative thoracic radiotherapy over 50Gy concurrent with platinum doublet chemotherapy.

Treatment comprised concurrent CRT and subsequent consolidation chemotherapy. Chemotherapy regimen was selected at investigator’s discretion. The doses and schedules were in accordance with the published reports [7,9-12]. All patients were treated with a linear accelerator photon beam of 4 MV or more. The primary tumor and involved nodal disease were to receive at least 60 Gy in 2-Gy fractions over 6 weeks. Our radiation technique was based on elective nodal irradiation. The radiation fields contained the primary tumor, ipsilateral hilum, and mediastinal nodal areas from the paratracheal to subcarinal lymph nodes. The contralateral hilum was not included, and the supraclavicular areas were not routinely treated.

### Assessment of outcomes and statistical analysis

Tumor response was classified in accordance with the Response Evaluation Criteria for Solid Tumors (RECIST), ver. 1.1. In almost all patients, tumor response was assessed every 2 courses of chemotherapy. After the treatment period, chest computed tomography (CT) was done every 2 to 3 months during the first year and at 3 to 6 month intervals thereafter. Positron emission tomography (PET) or PET-computed tomography (PET-CT) using 2-[^18^ F]-fluoro-2-deoxy-D-glucose (^18^ F-FDG) was performed at 6 to 12 month intervals if available. Magnetic resonance imaging (MRI) of the brain was performed only when clinical signs and symptoms suspicious for brain involvement were present. PFS was assessed from the first day of treatment with CRT to the earliest signs of disease progression as determined by CT or MRI imaging using RECIST criteria, or death from any cause.

The primary outcome of this study was to evaluate the surrogacy of ORR and PFS rate at 3-month intervals (from 9 to 24 months after the initiation of treatment) for the 5-year survival rate. Landmark analyses were performed to assess the association of these outcomes with the 5-year survival rate.

A p value of < 0.05 indicated statistical significance. The Kaplan-Meier method was used to estimate survival as a function of time. All the analyses were performed using JMP ver. 7 (SAS Institute Inc, USA) or R ver. 2. 15. 1. This retrospective analysis was approved by the institutional review board of Shizuoka Cancer Center.

## Results

A total of 159 consecutive patients were enrolled in this retrospective study. Baseline characteristics of the patients are summarized in Table 1. Median age was 64 years, 79% of patients were male, 75% were heavy smokers, 56% had an ECOG PS of 0, 53% had adenocarcinoma, and 54% were stage IIIB. Treatment characteristics are shown in Table 2. The most common regimens were carboplatin (CBDCA) plus paclitaxel, and cisplatin (CDDP) plus S-1 (46 patients each), and the third most frequent regimen was CDDP plus vinorelbine (VNR) (41 patients). The median radiation dose was 60 Gy (range, 52–74). The median follow-up time for censored patients was 57 months. At the time of analysis, 89 patients (56%) had died and 114 patients (72%) showed disease progression.

**Table 1:** Baseline characteristics

**Characteristic**	**N = 159**	
Age-year		
Median	64	
Range	40-75	
Sex-no. (%)		
Male	126	(79)
Female	33	(21)
Smoking status		
Non or light smoker	25	(16)
Heavy smoker	119	(75)
Unknown	15	(9)
ECOG performance status-no. (%)		
0	90	(57)
1	67	(42)
2	2	(1)
Histology-no. (%)		
ad	84	(53)
sq	54	(34)
Other	21	(13)
Clinical stage-no. (%)		
IIIA	86	(54)
IIIB	73	(46)

*Abbreviations:ECOG* Eastern Cooperative Oncology Group, *ad* adenocarcinoma, *sq* squamous cell carcinoma.

**Table 2:** Treatment characteristics

**Treatment**	**N = 159**	
Chemothrapy regimen-no. (%)		
CBDCA + PTX	46	(29)
CDDP + S-1	46	(29)
CDDP + VNR	41	(26)
MVP	14	(9)
CBDCA + CPT-11	5	(3)
CDDP + VP-16	4	(2)
CDDP + VNR + DE-766	3	(2)
RT dose-Gy		
Median	60	
Range	52-74	

*Abbreviations:CBDCA* carboplatin, *PTX* paclitaxel, *CDDP* cisplatin, *VNR* vinorelbine, *MVP* mitomycin, vindesine, and cisplatin, *CPT-11* irinotecan, *VP-16* etoposide, *RT* radiation therapy.

**Figure 1:**
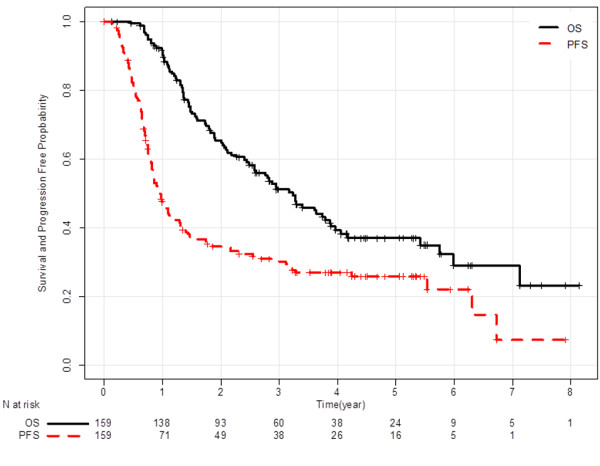
Kaplan-Meier-estimated PFS (dashed line) and OS curve (bold line) in LA-NSCLC patients treated with concurrent CRT (n = 159).

Complete response was observed in 6 patients, and 107 patients had partial response. Then, ORR was 72% (95% confidence interval [CI]: 65–78). Figure 1 shows Kaplan-Meier curves of PFS and OS. Median PFS was 12 months (95% CI: 10–14), and median OS was 39 months (95% CI: 30–46). Among 110 first relapse sites, 29 were loco-regional, 66 were distant, and 15 were both. Of 114 relapsed patients, 89 (78%) received subsequent chemotherapy, and 58 (51%) received third line chemotherapy. Six patients had *epidermal growth factor receptor (EGFR)* mutation, and they all were treated with gefitinib in a subsequent line. Six other patients demonstrated durable progression-free intervals (≥ 6 months) with EGFR-tyrosine kinase inhibitors, but their *EGFR* mutation status could not be assessed for lack of a sufficient specimen.

**Figure 2:**
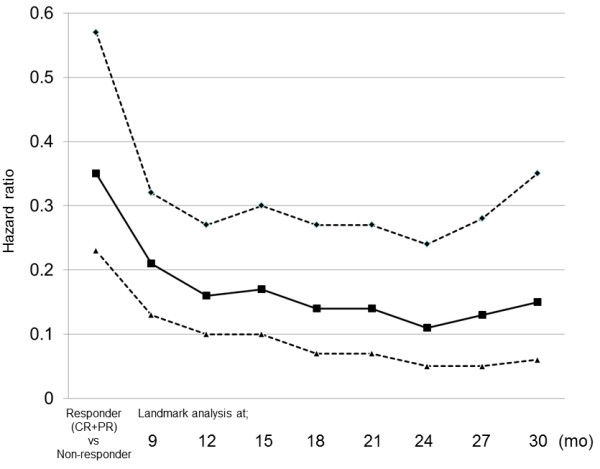
**Hazard ratio of landmark analysis at each time point.** Dashed lines indicate 95% confidence intervals. Abbreviations: CR, complete response; PR, partial response.

One hundred and forty-eight, 138, 121, 106, 101, 93, 87, and 79 patients who were alive at 9, 12, 15, 18, 21, 24, 27, and 30 months were included in the respective landmark analysis. The hazard ratio (HR) of patients who achieved progression-free to those who progressed at each landmark analysis is described in Figure 2. HR gradually decreased in accordance with progression-free interval extended, and reached the lowest level at 24 months (0.11; 95% CI: 0.05-0.24). Figures 1 and 2 suggest that an observational period of about 24 months is sufficient to detect almost all recurrences.

**Figure 3:**
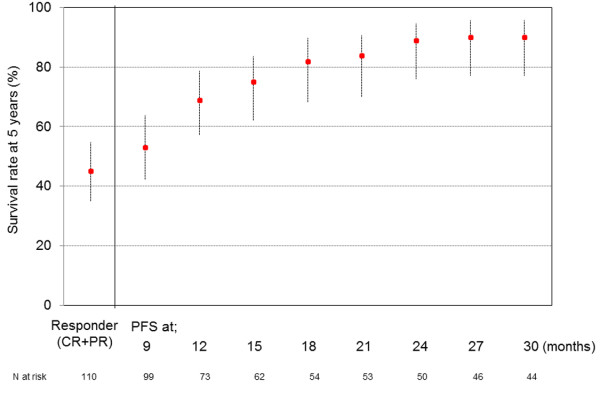
**Five-year survival rates of patients who achieved each outcome.** The bars indicate 95% confidence intervals.

Next, we examined the 5-year survival rates of patients who achieved response or progression-free at each time point. Among patients with complete response, or partial response, the 5-year survival rate was 45% (95% CI: 35–55) (Figure 3). The 5-year survival rates of patients who were progression free at each time point (3-months intervals from 9 to 30 months) were 53% (95% CI: 42–64), 69% (95% CI: 57–79), 75% (95% CI: 62–84), 82% (95% CI: 68–90), 84% (95% CI: 70–91), 89% (95% CI: 76–95), 90% (95% CI: 77–96), and 90% (95% CI: 77–96), respectively. The rate gradually increased in accordance with progression-free interval extended, and finally reached a plateau at 24 months. Patients who maintained progression-free intervals longer than 24 months had a 5-year survival rate of about 90%.

## Discussion

In this study, 159 LA-NSCLC patients treated with concurrent CRT were analyzed to evaluate the surrogacy of ORR and PFS rate at 3-month intervals for the 5-year survival rate. Kaplan-Meier curve of progression-free survival (Figure 1) and HR of landmark analysis at each time point (Figure 2) suggest that most of progression occurred in the first 2 years. Patients who maintained progression-free intervals longer than 2 years had a 5-year survival rate of approximately 90%, and the rate did not increase thereafter (Figure 3).

Although ORR could be assessed in the early period of CRT, its surrogacy for the 5-year survival rate has not been fully evaluated. McAleer et al., did a combined analysis of two RTOG studies with CRT [13]. They reported that response to induction chemotherapy was a possible predictor of long survival (p = 0.06). Kim et al., also reported that responders demonstrated 5-fold long term survival compared with non-responders among LA-NSCLC patients treated with CRT [14]. However, in McAleer’s report, Kaplan-Meier curves of OS revealed that 90% of responders died within 4 years. Furthermore, Kim’s report was premature because the median follow-up time was only 489 days. Our analysis, with a longer follow up period, demonstrated that the ORR was not a favorable surrogate marker for the 5-year survival rate.

With regard to median PFS, Mauguen et al., conducted a meta-analysis of LA-NSCLC. They found a very good correlation between median PFS and OS both at the individual level and trial level (ρ^2^ range; 0.77-0.85, R2 range; 0.89-0.97, respectively) [15]. However, it is worth noting that their analysis contained relatively old trials. The median survival time of 15 months reported by Mauguen et al. was much shorter than that in a recent phase III trial, which reported a median survival time of 29 months [16]. This prolongation of survival may account for the development of post progression therapy, as the median PFS did not differ between the 2 reports. This might be a cause for concern about the relationship between median PFS and OS. In fact, our analysis showed that the 5-year survival rates in patients who were disease free at 9–12 months were only 53-69%. The rate gradually increased in accordance with progression-free interval extended, and reached a plateau at 90% after 24 months. This suggests that longer progression-free period, not median PFS, is required to identify cured patients.

The present study has several limitations. First, this study contained various chemotherapy regimens, and the timing of evaluatton depended on investigators because this was a retrospective study. Second, efficacy results were slightly better than previous reports. In our analysis, about 70% of patients were screened with PET (or PET-CT) at diagnosis, and 3-dimensional conformal radiation therapy was adopted in all cases. These contributed to accurate staging, and proper radiation therapy. In addition, the proportion of patients who received post progression therapy was very high (approximately 80%).

## Conclusion

Our study suggests that PFS at 2 years could be a reliable surrogate endpoint for 5-year survival rate in LA-NSCLC patients treated with concurrent CRT. Further analysis is warranted using prospective datasets.

## Competing interest

The authors declare that they have no competing interests.

## Authors’ contributions

HA contributed to the drafting of this manuscript and data collection, and KM, and TN contributed to the study design and statistical analysis. HI, TS, TT, HK, HM, ME, HH, TT, and NY contributed to analysis of the data and interpretation of the findings. All authors have read and approved of the submission of the final manuscript.

## Pre-publication history

The pre-publication history for this paper can be accessed here:

http://www.biomedcentral.com/1471-2407/14/18/prepub
